# Changes in Adolescent Birth Rates within Appalachian Subregions and Non-Appalachian Counties in the United States, 2012–2018

**DOI:** 10.13023/jah.0401.05

**Published:** 2022-02-13

**Authors:** Nathan Hale, Kathleen Tatro, Sylvester Olubolu Orimaye, Michael Smith, Michael Meit, Kate E. Beatty, Amal Khoury

**Affiliations:** East Tennessee State University; East Tennessee State University; East Tennessee State University; East Tennessee State University; East Tennessee State University; East Tennessee State University; East Tennessee State University

**Keywords:** Appalachia, adolescent births, economic vulnerability, adolescent health

## Abstract

**Background:**

Adolescent births are associated with numerous challenges. While adolescent birth rates have declined across the U.S., disparities persist, and little is known about the extent to which broader declines are seen within Appalachia.

**Purpose:**

The purpose of this study was to examine the extent to which adolescent birth rates have declined across the subregions of Appalachia relative to non-Appalachia.

**Methods:**

We conducted a retrospective study of adolescent birth rates between 2012 and 2018 using county-level vital records data. Differences were examined across the subregions of Appalachia and among non-Appalachian counties. Multiple regression models were used to examine changes in the rate of decline over time, adjusting for additional covariates of relevance.

**Results:**

About 13.4% of all counties in the U.S. are within the Appalachian region. The rate of adolescent births decreased by 12.6 adolescent births per 1,000 females between 2012 and 2018 across the U.S. While all regions experienced declines in the rate of adolescent births, Central Appalachia had the largest reduction in adolescent births (18.5 per 1,000 females), which was also noted in the adjusted models when compared to the counties of non-Appalachia (b= −5.78, CI: −9.58, −1.97). Rates of adolescent birth were markedly higher in counties considered among the most socially and economically vulnerable.

**Implications:**

This study demonstrates that the rates of adolescent births vary across the subregions of Appalachia but have declined proportional to rates in non-Appalachia. While adolescent birth rates remain higher in select subregions of Appalachia compared to non-Appalachia, the gap has narrowed considerably.

## INTRODUCTION

Adolescent births in the U.S. remain an important public health issue. The majority of births to adolescents are either mistimed or unwanted and pose additional educational, economic, and health-related challenges for young mothers relative to their peers.^1^,^2^ Furthermore, poor obstetric and infant health outcomes are more common among births to adolescent mothers.^2^ Adolescent births are also costly, totaling $37 billion annually in health-related and social services spending.^2^

Adolescent birth rates have steadily declined in the U.S. over the past three decades, reaching a record low of fewer than 18 births per 1,000 females between 15 and 19 years of age in 2018.^1^,^2^ These declines have been observed across all racial/ethnic population groups. While a broad array of factors contributes to observed declines, delayed sexual initiation and the increased use of contraceptives, specifically the more effective long-acting reversible contraceptives (LARCs), are two important factors associated with the marked decrease in adolescent births.^3^,^4^ These factors suggest an increasing level of reproductive autonomy among adolescents.

Despite the continued progress, the rate of adolescent births remains higher in the U.S. than in other industrialized nations, and adolescent birth rates for Hispanic and non-Hispanic black persons remain two times higher than observed rates among non-Hispanic white teens.^3^–^5^ Socioeconomic vulnerability is an important factor associated with increased adolescent births.^6^,^7^ Research has also noted geographic differences in the rate of adolescent births, with higher rates among adolescents residing in rural counties relative to their urban counterparts.^2^,^8^,^9^ Additional studies have examined the intersection of geographic and socioeconomic vulnerability, noting that adolescent births are highest in rural counties that are also among the most economically disadvantaged.^6^,^9^

Appalachia consists of mostly rural counties that are disproportionately represented among persistent poverty counties, particularly Central Appalachia.^10^,^11^ Health disparities, including adolescent births, in Appalachia are well documented.^11^,^12^ Geographic isolation, limited economic mobility, and persistent challenges in access to providers and affordable preventive health services contribute to observed disparities in this region relative to what is observed nationally.^11^,^12^ The provision of contraceptive counseling and reproductive health services through federally funded clinics in rural communities, such as Appalachia, is limited.^13^ Access to comprehensive sexual health education may also be limited, both potentially contributing to higher rates of adolescent births in Appalachia.^14^

While disparities related to adolescent births have been documented in Appalachia,^2^,^11^,^12^ there is a paucity of research examining changes over time and specifically within the subregions of Appalachia and how these compare to non-Appalachia. Appalachia is comprised of 420 counties stretching from Mississippi to New York.^11^ While the counties of Appalachia share a similar geographic designation—characteristics of regional populations and available health and healthcare resources vary. Important regional differences and subsequent strategies for addressing longstanding disparities may be missed when examining Appalachia as a whole.

The purpose of this study is two-fold: examine differences in adolescent birth rates between Appalachian subregions and examine how changes in adolescent birth rates in the subregions of Appalachia compare to what was observed among non-Appalachian counties during the same period. While adolescent birth rates in Appalachia have been historically higher than what was observed outside of Appalachia, the rate of change over time has not been examined. The extent to which observed differences have persisted or narrowed over time relative to national trends remains unknown.

## METHODS

### Study Design and Data Sources

A retrospective study of adolescent birth rates between 2012 and 2018 was conducted using county-level vital records data. These years capture the immediate post-Affordable Care Act (ACA) period with favorable reproductive health policies. County-level birth rates from the National Center for Health Statistics (NCHS) were linked with the American Community Survey (ACS) 5-year population estimates from the Census Bureau. Additional county-level variables of interest included Health Professional Shortage Area (HPSA) categories from the Area Health Resource File (AHRF) and an index-based economic classification from the Appalachian Regional Commission (ARC). Data for all U.S. counties were analyzed, which includes 420 Appalachian counties and 2,723 non-Appalachian counties.^11^

### Outcome Measures and Covariates of Interest

Adolescent birth rates were the primary outcome examined. Consistent with previous research,^2^,^4^ adolescent birth was identified as the number of pregnancies with live birth outcomes among females aged 15–19 in each county per year. While unintended pregnancies among adolescents are important to consider, abortion and fetal loss data were not available, and the study therefore focuses on live births to adolescents as the primary outcome. The subregions of Appalachia, as designated by the ARC, is of primary interest in this study. Given this study is focused on changes in adolescent birth rates, time is also a primary independent variable. A dichotomous measure indicating baseline (2012) and end line (2018) was included in the analysis.

To account for the influence of socioeconomic vulnerabilities that are more common among the counties of Appalachia that may also explain observed differences in adolescent birth rates beyond geography and consistent with previous literature, the ARC county economic status was included in the analysis.^15^ The ARC economic classification combines 3-year average unemployment rate, poverty rate, and per capita market income which are subsequently ordered as a five-level categorical variable ranking counties as attainment, competitive, transitional, at risk, or distressed. Counties identified as attainment and competitive represent the top 25% of U.S. counties, whereas those identified as at risk or distressed represent the bottom 25% of counties.^15^ In order to assess county-level access to healthcare services HPSA classifications were included. Further, to account for the varying degrees of financial resources needed to access preventative services the percent of females under 18 years enrolled in Medicaid and the percentage of residents under 65 years without any insurance were also included. Additional county-level demographic measures were included reflecting the proportion of white, black, Hispanic, other population groups (Asian, Native Hawaiian/Pacific Islander, American Indian), and non-English speaking populations.^10^

### Statistical Analysis

One-way analysis of variance (ANOVA) was utilized to examine differences in the characteristics of the Appalachian subregions by the covariates of interest. Bivariate differences in adolescent birth rates between 2012 and 2018 were examined for each Appalachian subregion using the unadjusted regression model and predicted means of adolescent birth rates for both years (2012 and 2018). The absolute difference between 2012 and 2018 was examined to identify initial variation in adolescent birth rates at baseline and the rate of change for each Appalachian subregion over time, relative to what was observed among non-Appalachian counties. Generalized Estimating Equations (GEE) were used for the multiple linear regression model. GEE provides population-averaged estimates of the adolescent birth rates, which is relevant to estimating reliable adolescent birth rates of each Appalachian subregion with multiple counties.

Moreover, GEE accounts for intra-subject (intra-county) correlation given that adolescent birth rates potentially vary for each county over a period of years. A regression-based difference-in-difference approach was taken for the adjusted model. Time was interacted with the categorical measure denoting Appalachia designation (non-Appalachia, Northern, North Central, Central, South Central, and Southern Appalachia) in a single model to examine differences in the rate of change between 2012 and 2018 while adjusting for additional covariates of interest. Adolescent birth rates among non-Appalachian counties at baseline were the reference value. Coefficients (b) reflect population-averaged differences in adolescent birth rates relative to the reference value.

Predicted means and 95% confidence intervals were derived from the adjusted model to further examine longitudinal differences in the rate of change between 2012 and 2018. Importantly, the study accounts for the precision of the estimated adolescent birth rates across the Appalachian subregions by including a weighting factor in all analyses. Since the total and sample female populations in each county within each Appalachian subregion varies with potentially smaller counties having higher adolescent birth rates and vice-versa, the weighting factor was estimated as the ratio of the sample population of females aged 15–19 in each county to the total female population in each county. The study was reviewed by the East Tennessee State University (ETSU) Institutional Review Board (IRB) and deemed non-human subjects research. All data management and analyses were performed using Statistical Analysis Software (SAS) version 9.4 (SAS Institute Inc., Cary, NC, USA).

## RESULTS

About 13.4% of all counties in the U.S. are within the Appalachian region (Table 1). Statistically significant differences between the Appalachian subregions and the non-Appalachian region were observed for all the select characteristics including economic classification, access to health care, race/ethnicity, and health insurance.

Among the non-Appalachian counties, 21.8% were designated as at risk or distressed, compared to 42.3% in Southern Appalachia, 30.5% in South Central Appalachia, 92.6% in Central Appalachia, 58.7% in North Central Appalachia, and 8.1% in Northern Appalachia (p<0.001). Whole and partly HPSA designated counties represent 87.4% of non-Appalachia compared to 88.0% in Southern Appalachia, 84.2% in South Central Appalachia, 85.9% in Central Appalachia, 89.1% in North Central Appalachia, and 89.5% in Northern Appalachia (p<0.001).

On average, white persons make up 91.5% of the female population across the five Appalachian subregions compared to the 83.2% of the female population in non-Appalachia. On average, a higher proportion of adolescents across the Appalachian subregions were enrolled in Medicaid (47.2%), compared to 40.0% across the non-Appalachian counties. The proportion of residents under age 65 without any health insurance was significantly higher in Southern Appalachia (16.8%) compared to 15.1% across non-Appalachian counties, 16.5% in South Central Appalachia, 13.4% in Central Appalachia, 12.8% in North Central Appalachia, and 9.9% in Northern Appalachia.

The adolescent birth rate was markedly higher in Central Appalachia and lower in Northern Appalachia at baseline than what was observed among non-Appalachian counties and the other Appalachian subregions (Table 2). A similar distribution was noted at the end of the study period.

While all regions experienced declines in the rate of adolescent births, the level of change was not uniform. The rate of adolescent births decreased by 12.5 adolescent births per 1,000 females between 2012 and 2018 in non-Appalachia. Among the subregions of Appalachia reductions in the adolescent birth rate per 1,000 females ranged from 9.8 (Northern Appalachia) to 18.5 (Central Appalachia). Compared to the non-Appalachian region, Central Appalachia had the largest reduction in adolescent birth rates over the seven-year period.

When adjusted for select characteristics, longitudinal differences in adolescent birth rates were noted across the Appalachian subregions (Table 3). Notably, the reductions in adolescent birth rates among the counties of Central Appalachia were markedly different than what was observed among the counties of non-Appalachia (b= −5.78, CI: −9.58, −1.97). Among the additional covariates of interest, adolescent birth rates were higher among counties classified as transitional (b=5.86, CI: 4.08, 7.64) and markedly higher among counties at risk (b=8.62, CI: 6.22, 11.03) or distressed (b=11.17, CI: 8.23, 14.10) compared to the reference group of attainment.

Females aged 15–19 years of Hispanic ethnicity (b=0.32, CI: 0.23, 0.40); females aged 15–19 in other racial classifications (b=0.24, CI: 0.09, 0.38); the percentage of females under 18 years enrolled in Medicaid (b=0.21, CI: 0.15, 0.26); and the percentage of uninsured residents under age 65 (b=0.58, CI: 0.48, 0.68) were significantly associated with higher rates of adolescent births. However, the percentage of non-English speaking individuals (b= −0.55, CI: −0.76, −0.34) was associated with lower adolescent birth rates.

The adjusted predicted means of adolescent birth rates by Appalachia subregion from the adjusted regression models are provided in Table 4. Statistically significant differences between 2012 and 2018 were noted across all the Appalachian subregions and the non-Appalachian region. A larger decrease in the predicted mean of adolescent birth rates was observed for Central Appalachia (16.9 births per 1,000 females aged 15–19 years) when compared to non-Appalachian counties (11.1 births per 1,000 females aged 15–19).

## IMPLICATIONS

The study finds that adolescent birth rates are higher in most Appalachian subregions relative to what is observed in non-Appalachia. Notably, adolescent birth rates in Central Appalachia were markedly higher than what was observed among non-Appalachian counties at baseline. However, the rate of decline in adolescent birth rates observed among the counties of Central Appalachia over the 7-year period was considerably steeper than what was observed among non-Appalachian counties and other Appalachian subregions. While adolescent birth rates remain higher in Central Appalachia, the gap has narrowed considerably over time.

While the overall adolescent birth rate decreased over time, rates remained much higher among counties demonstrating socioeconomic vulnerability. Adolescent birth rates among the most economically distressed counties were markedly higher than what was observed among the more affluent counties. Socioeconomic vulnerability, as reflected in the ARC economic status classification, is an important factor influencing health outcomes and the counties of Appalachia are disproportionately represented among the at risk and distressed communities—particularly Central Appalachia.^10^,^11^,^15^ The ARC has noted that the majority of the Central Appalachian counties had a lower median household income than the national average.^10^ Continued investments in education, technology, infrastructure and economic development within Appalachia are critical for addressing the factors associated with increased community deprivation and poor health outcomes.

The study notes that the percentage of uninsured residents below age 65 was associated with an increased rate of adolescent birth. Insurance coverage is important for enabling access to effective contraception and contributes to positive health outcomes.^7^,^17^–^19^ While not independently associated with adolescent births, HPSA designation was also more common among Appalachian counties. Taken collectively, these findings underscore the potential impact of structural, financial, and geographic barriers to accessing providers and contraceptive services among vulnerable populations, such as the Appalachian region.^17^,^20^

Increased Medicaid enrollment among females aged less than 19 years was also associated with increased adolescent birth rates. With the exception of Northern Appalachia, Medicaid enrollment among individuals aged 19 years and under was far more common in Appalachian counties than non-Appalachia. While Medicaid enrollment may provide financial access to services, it may also reflect increasing socioeconomic vulnerability within populations. Recent research has noted that Medicaid expansion has improved access to contraception, specifically noting that the use of most effective contraceptive methods increased in expansion states compared to states that did not expand Medicaid.^21^ Importantly, states encompassing Central Appalachia, which include parts of Kentucky, Virginia, and West Virginia, were among those expanding state Medicaid programs. The extent to which Medicaid expansion may explain observed changes in adolescent births within Central Appalachia remains an important area for future research.

It is worth noting that adolescent birth rates in Northern Appalachia were lower than what was observed among non-Appalachian counties and other Appalachian subregions at baseline. Northern Appalachia also experienced the smallest decline in adolescent births over the 7-year period, which is largely expected given the lower baseline value. Northern Appalachia also had fewer counties among the most economically distressed, fewer counties designated as Whole Health Professional Shortage Areas, lower proportions of populations of uninsured individuals and lower proportions of individuals aged less than 19 years enrolled in Medicaid than what is observed among non-Appalachia counties and the other Appalachian subregions. These findings underscore that Appalachia is not a homogenous population. Considering Appalachia as a whole, while important, may mask important health disparities and variation in observed outcomes within the subregions.

This study is not without limitations. Adolescent birth rates have fallen over multiple decades in the U.S. This study examines a shorter time window within this broader context. It is possible that observed changes during the study period differ within a larger context spanning multiple decades or may not represent broader changes over a longer period. The cause of differences in adolescent birth rates between Appalachia and non-Appalachian counties and within the different Appalachian subregions was not examined specifically and remains an active area for future research. Having a better understanding of the extent to which changes in access to preventive clinical services, sexual behaviors, investments in pregnancy prevention, and other contextual factors is needed and would greatly inform future actionable steps to sustain observed improvements. Also, other sources of variation such as nationwide healthcare policies, state-level expansion of Medicaid programs, and regionally tailored healthcare programs were not considered in this study. Examining the longitudinal effects of state and national policies focused on access to reproductive health services and the influence of varying underlying economic vulnerabilities within the subregions of Appalachia is warranted.^22^,^23^

Although potential for missing data, misclassification, and changes to data elements remain a salient issue with any secondary data sources, this study uses large, well-established data source to examine an important public health issue. While we were not able to understand the contextual factors contributing to observed differences in this study, we were able to use these data to uncover important variation in adolescent birth rates. The finding that the overall rate of birth to adolescents is decreasing not just nationally, but within the subregions of Appalachia is encouraging. The underlying reasons why the rate of decline in Central Appalachia was steeper, despite less favorable social, economic, and healthcare-access-related characteristics, is not well understood and needs further attention. The study spans a time frame that includes an unprecedented federal investment in adolescent pregnancy and innovative federal partnerships to focus on the issue and scale up evidence-based interventions, following the ACA.^24^ These programs coupled with expanded access to health insurance and removing cost barriers for preventive health services, including contraception, are likely important factors that may contribute to observed declines. The study also includes a period of program cuts and shifting program focus, undermining access to evidence-based programs and clinical services for adolescents.^25^ Ensuring that adolescents in Appalachia have access to the important educational and clinical services needed to make decisions related to reproductive health and family planning should be a continued priority.

## CONCLUSION

This study examined changes in adolescent birth rates over time among the subregions of Appalachia, relative to non-Appalachia. Available evidence indicates that this is the first study that has examined this issue longitudinally within the subregions of Appalachia. Its findings unmask important variation in adolescent birth rates among the subregions of Appalachia. It finds that adolescent birth rates were higher among most Appalachian subregions relative to non-Appalachian counties. While the overall adolescent birth rate was much higher in Central Appalachia at baseline, significant progress has been made in this subregion with notable declines during the study period. This is encouraging for clinical practitioners at the front lines and affirms the impact of the reproductive health services they provide to adolescents. This is also encouraging for public health practitioners involved in health education and promotion in communities in Central Appalachia. The study also found that community deprivation and limited access to healthcare providers and services were associated with increasing rates of adolescent births, and these factors were more common among the counties of Appalachia. The study has important public health and health policy implications. Its findings may support the efforts of practitioners and advocates seeking to expand access to care in their communities. These findings can be also used to further target public health interventions and provide a more nuanced perspective on health outcomes within the Appalachian subregions.

**SUMMARY BOX**

**What is already known about this topic?** While adolescent birth rates have been declining over the last several decades, geographic variations continue to persist, particularly in rural and socioeconomically disadvantaged regions. Adolescent births are an important public health issue as they pose educational, economic, and health-related challenges for teen mothers and their infants.

**What is added by this report?** Available evidence indicates that this is the first study to examine adolescent birth rates longitudinally within the subregions of Appalachia. The findings unmask important variation in adolescent birth rates among the subregions of Appalachia. This study demonstrates that while adolescent birth rates generally remain higher in Appalachia than in non-Appalachian regions, the gaps among U.S. regions have narrowed substantially.

**What are the implications for future research?** The identification of subregional variation in adolescent birth rates in Appalachia provides a more nuanced view of important differences and changes over time. This research further provides evidence for the importance of increasing access to reproductive healthcare services and comprehensive sexual health education in Appalachia and other rural communities. Further understanding the extent to which changes in access to preventive clinical services, sexual behaviors, investments in pregnancy prevention, and other contextual factors contributed to observed changes over a longer duration is needed and would greatly inform future actionable steps to sustain observed improvements.

## Figures and Tables

**Table 1:** Characteristics of U.S. counties by Appalachian subregion from 2012–2018, mean (95% CI)

County Characteristics	Non-Appalachia (n= 2,723)	Southern Appalachia (n=104)	South Central Appalachia (n= 85)	Central Appalachia (n= 82)	North Central Appalachia (n= 63)	Northern Appalachia (n= 86)
**ARC Economic Classification**						
Attainment (%)*	11.44 (10.98, 11.89)	1.93 (0.93, 2.93)	1.17 (0.30, 2.04)	0 (0, 0)	0 (0, 0)	0 (0, 0)
Competitive (%)*	16.97 (16.44, 17.51)	0.96 (0.25, 1.67)	2.34 (1.12, 3.56)	0 (0, 0)	3.18 (1.54, 4.83)	5.81 (3.94, 7.69)
Transitional (%)*	49.74 (49.03, 50.46)	54.80 (51.18, 58.42)	65.89 (62.07, 69.71)	7.33 (5.20, 9.47)	38.07 (33.52, 42.62)	86.06 (83.29, 88.84)
At Risk (%)*	13.26 (12.78, 13.74)	35.56 (32.07, 39.04)	25.90 (22.37, 29.43)	26.81 (23.18, 30.45)	36.49 (31.98, 41.00)	6.98 (4.94, 9.02)
Distressed (%)*	8.58 (8.18, 9.98)	6.75 (4.92, 8.58)	4.69 (2.99, 6.40)	65.85 (61.95, 69.74)	22.25 (18.36, 26.15)	1.14 (0.29, 2.00)
**HPSA Categories**						
None Designated (%)	12.65 (12.18, 13.13)	11.96 (9.60, 14.32)	15.79 (12.86, 18.73)	14.14 (11.28, 17.00)	10.95 (8.03, 13.88)	10.47 (8.02, 12.92)
Parts Designated (%)*	60.64 (59.94, 61.34)	57.16 (53.56, 60.76)	68.80 (65.07, 72.53)	53.84 (49.75, 57.93)	66.83 (62.42, 71.24)	82.86 (79.85, 85.88)
Whole Designated (%)*	26.71 (26.08, 27.34)	30.88 (27.51, 34.24)	15.41 (12.50, 18.32)	32.02 (28.20, 35.85)	22.22 (18.33, 26.12)	6.67 (4.67, 8.67)
**Populations, females aged 15–19**						
White (%)*	83.19 (82.95, 83.44)	78.02 (76.79, 79.25)	92.76 (92.27, 93.25)	96.74 (96.57, 96.91)	95.93 (95.62, 96.24)	93.95 (93.51, 94.39)
Black (%)*	9.74 (9.53, 9.96)	18.41 (17.15, 19.67)	3.70 (3.36, 4.04)	1.92 (1.77, 2.06)	2.51 (2.28, 2.74)	3.73 (3.38, 4.09)
Hispanic (%)*	9.99 (9.78, 10.19)	5.21 (4.83, 5.60)	3.94 (3.73, 4.15)	1.35 (1.28, 1.43)	1.21 (1.12, 1.29)	2.48 (2.29, 2.66)
Other (%)*	4.39 (4.26, 4.53)	1.72 (1.59, 1.85)	2.12 (1.81, 2.44)	0.79 (0.75, 0.84)	1.13 (1.04, 1.21)	1.52 (1.39, 1.64)
Non-English-Speaking Residents (%)*	3.70 (3.63, 3.78)	2.52 (2.31, 2.72)	1.76 (1.65, 1.86)	0.63 (0.59, 0.68)	0.65 (0.59, 0.72)	1.73 (1.54, 1.91)
**Insurance Coverage**						
Female < 18 years using Medicaid (%)*	39.96 (39.75, 40.16)	47.31 (46.51, 48.11)	45.26 (44.39, 46.13)	56.91 (56.14, 57.68)	48.54 (47.63, 49.44)	37.93 (37.34, 38.51)
Residents under 65 without insurance (%)*	15.05 (14.95, 15.15)	16.84 (16.52, 17.16)	16.47 (16.09, 16.85)	13.44 (13.18, 13.71)	12.75 (12.45, 13.05)	9.94 (9.65, 10.24)

* p<0.0001

**Table 2:** Bivariate association between adolescent birth rate per 1,000 females aged 15–19 by region

Region	2012	2018	Absolute Difference
Non-Appalachia*	36.19	23.64	−12.55
Southern Appalachia*	42.97	28.53	−14.44
South Central Appalachia*	39.62	26.26	−13.36
Central Appalachia*	55.31	36.78	−18.53
North Central Appalachia*	42.85	28.62	−14.23
Northern Appalachia*	27.38	17.57	−9.81

* p<0.0001

**Table 3:** Adjusted geographic differences in adolescent birth rates by ARC economic classification and HPSA

Variable	Coefficient (95% CI)
**Subregion**	
Non-Appalachia	Ref
Southern Appalachia	3.11 (0.44, 5.77)
South Central Appalachia	1.97 (−0.69, 4.64)
Central Appalachia	13.60 (9.56, 17.64)
North Central Appalachia	5.41 (2.10, 8.71)
Northern Appalachia	−3.57 (−5.90, −1.23)
**Year**	
2012	Ref
2018	−11.08 (−11.93, −10.24)
**Subregion x Year**	
Non-Appalachia x 2012	Ref
Southern Appalachia x 2018	−1.97 (−4.15, 0.20)
South Central Appalachia x 2018	−0.84 (−3.17, 1.49)
Central Appalachia x 2018	−5.78 (−9.58, −1.97)
North Central Appalachia x 2018	−1.10 (−4.15, 1.95)
Northern Appalachia x 2018	2.80 (0.97, 4.62)
**ARC Economic Classification**	
Attainment	Ref
Competitive	1.67 (−0.03, 3.37)
Transitional	5.86 (4.08, 7.64)
At Risk	8.62 (6.22, 11.03)
Distressed	11.17 (8.23, 14.10)
**HPSA**	
No shortage	Ref
Partial shortage	−0.71 (−1.76, 0.35)
Whole shortage	0.65 (−0.73, 2.03)
**Populations, females aged 15–19**	
White (%)	0.10 (−0.03, 0.23)
Black (%)	0.18 (0.04, 0.32)
Hispanic (%)	0.32 (0.23, 0.40)
Other (%)	0.24 (0.09, 0.38)
Non-English speaking (%)	−0.55 (−0.76, −0.34)
Females <18 years using Medicaid (%)	0.21 (0.15, 0.26)
Residents <65 without insurance (%)	0.58 (0.48, 0.68)

**Table 4:** Predicted adolescent birth rate per 1,000 females aged 15–19 by region

Region	2012	2018	Absolute Difference
Non-Appalachia*	35.73	24.64	−11.09
Southern Appalachia*	38.84	25.77	−13.07
South Central Appalachia*	37.70	25.78	−11.92
Central Appalachia* †	49.33	32.46	−16.87
North Central Appalachia*	41.14	28.95	−12.19
Northern Appalachia*	32.16	23.87	−8.29

* p<0.0001

† Significantly different change than non-Appalachia (p<0.05)
